# Chamomile as a potential remedy for obesity and metabolic syndrome

**DOI:** 10.17179/excli2021-4013

**Published:** 2021-07-26

**Authors:** Maria M. Bayliak, Tetiana R. Dmytriv, Antonina V. Melnychuk, Nadia V. Strilets, Kenneth B. Storey, Volodymyr I. Lushchak

**Affiliations:** 1 Department of Biochemistry and Biotechnology, Vasyl Stefanyk Precarpathian National University, 57 Shevchenko Str., Ivano-Frankivsk, 76018, Ukraine; 2 Institute of Biochemistry, Carleton University, 1125 Colonel By Drive, Ottawa, Ontario K1S 5B6, Canada; 3 I. Horbachevsky Ternopil National Medical University, 46002, Ternopil, Ukraine; 4 Research and Development University, Shota Rustaveli Str., 76018, Ivano-Frankivsk, Ukraine

**Keywords:** Chamomile, polyphenols, inflammation, pro-oxidant, Nrf2, PPARgamma

## Abstract

Obesity is an increasing health concern related to many metabolic disorders, including metabolic syndrome, diabetes type 2 and cardiovascular diseases. Many studies suggest that herbal products can be useful dietary supplements for weight management due to the presence of numerous biologically active compounds, including antioxidant polyphenols that can counteract obesity-related oxidative stress. In this review we focus on *Matricaria chamomilla*, commonly known as chamomile, and one of the most popular medicinal plants in the world. Thanks to a high content of phenolic compounds and essential oils, preparations from chamomile flowers demonstrate a number of pharmacological effects, including antioxidant, anti-inflammatory, antimicrobial and sedative actions as well as improving gastrointestinal function. Several recent studies have shown certain positive effects of chamomile preparations in the prevention of obesity and complications of diabetes. These effects were associated with modulation of signaling pathways involving the AMP-activated protein kinase, NF-κB, Nrf2 and PPARγ transcription factors. However, the potential of chamomile in the management of obesity seems to be underestimated. This review summarizes current data on the use of chamomile and its individual components (apigenin, luteolin, essential oils) to treat obesity and related metabolic disorders in cell and animal models and in human studies. Special attention is paid to molecular mechanisms that can be involved in the anti-obesity effects of chamomile preparations. Limitation of chamomile usage is also analyzed.

## Abbreviations

AMPK 5' adenosine monophosphate-activated protein kinase

AP-1 activator protein-1

FOXO1 forkhead box O protein 1

GLUT-4 glucose transporter type 4

HFD high fat diet

FFAs free fatty acids

LDL low density lipoproteins

NF-κB nuclear factor-κB

Nrf2 nuclear factor erythroid 2 related factor 2

PPARγ peroxisome proliferator-activated receptor gamma

ROS reactive oxygen species

STZ streptozotocin

TAG triacylglycerides

TNFα tumor necrosis factor alpha

## Introduction

Obesity is a non-infectious chronic metabolic disorder that has become a medical and social issue of 21^st^ century (Bray et al., 2017[13]). Intensive fat accumulation is accompanied by inflammation and oxidative stress in adipose tissue which further aggravate metabolic disturbances and provoke obesity-related cardiovascular, cerebrovascular and metabolic complications (Fernández-Sánchez et al., 2011[35]; Kanda et al., 2011[58]; Matsuda and Shimomura, 2013[87]; Catrysse and van Loo, 2017[16]; Bayliak and Abrat, 2020[10]). Metabolic alterations include increased levels of blood triacylglyce-ride (TAG), cholesterol and low-density lipoproteins with decreased levels of high-density lipoproteins, hypertension and impaired glucose tolerance. These signs together characterize metabolic syndrome, which is currently considered as a chronic metabolic disease causing increased risk of concomitant pathologies including insulin resistance, diabetes 2 type and liver disease (Grundy, 2004[47]; Laclaustra et al., 2007[68]; Saklayen, 2018[112]). 

Global anti-obesity strategies are focused on dietary and lifestyle changes, i.e., change the energy balance so that calories spent prevail over calories consumed along with an increase in physical activity (Mehta et al., 2021[89]). Natural product-based drug interventions are also considered to be an important approach for obesity management (González-Castejón et al., 2011[46]; Mehta et al., 2021[89]). Various dietary bioactive compounds can be useful in the prevention of obesity-associated metabolic disorders via targeting of digestion processes, different stages of adipocyte proliferation and differentiation, and molecular and metabolic pathways related to obesity (González-Castejón et al., 2011[46]).

*Matricaria chamomilla* (synonym: *Matricaria recutita*), commonly known as chamomile, is one of the most popular medicinal plants in the world (Singh et al., 2011[121]; Srivastava et al., 2010[124]; Raal et al., 2012[106]). Preparations from chamomile flowers have antioxidant, anti-inflammatory, antiseptic, antispasmodic and sedative effects (McKay and Blumberg, 2006[88]; European Medicines Agency, 2015[29]), and contain a number of biologically active compounds, including essential oils (*e.g.* chamazulene, α-bisabolol and spiroethers) and phenolic compounds - phenolic acids, coumarins and flavonoids (Mulinacci et al., 2000[93]; McKay and Blumberg, 2006[88]; Haghi et al., 2014[50]). 

*In vitro* and *in vivo* studies have shown that polyphenols have protective effects against oxidative stress-related diseases, including metabolic syndrome and obesity (Zang et al., 2006[145]; Zhang et al., 2015[150]; Pallauf et al., 2017[98]; Gómez-Zorita et al., 2017[45]; Kwon and Choi, 2018[66]; Gentile et al., 2018[42]). In line with this, chamomile extracts were found to protect against oxidative damage associated with obesity (Jabri et al., 2016[56], 2020[54]; Zemestani et al., 2016[146]). The antioxidant effects seem to attribute not only phenolic compounds but also to essential oils (Querio et al., 2018[105]). Modulation of signaling pathways related to energy metabolism, inflammation, stress responses and adipogenesis were also found under treatment with chamomile extracts or its individual components (Weidner et al., 2013[140]; Pallauf et al., 2017[98]; Lu et al., 2019[75]; Ma et al., 2020[80]). Some studies report that chamomile inhibits carbohydrate digestion and glucose absorption in the intestine (Villa-Rodriguez et al., 2018[136]; de Franco et al., 2020[21]). In addition, chamomile extract and its individual flavonoids show hypoglycemic and hypocholestrolemic effects (Panda and Kar, 2007[99]; Kato et al., 2008[59]; Najla et al., 2012[95]; Zhang et al., 2016[147]; Zemestani et al., 2016[146]). Thus, chamomile might be a potentially effective anti-obesity agent and remedy for metabolic syndrome treatment. In this paper, current data on the anti-obesity potential of chamomile and its individual compounds are summarized with analysis of potential mechanisms of protective action of therapeutically interesting active compounds isolated from chamomile flowers. 

## Obesity and Metabolic Syndrome

### Causes and complications

Obesity is a metabolic condition characterized by an excess of fat in the subcutaneous tissue and other tissues of the body, which occurs when food/energy intake exceeds food/energy utilization. Excessive caloric intake combined with low physical activity are considered to be the main cause of obesity worldwide (Bray et al., 2017[13]; Bayliak et al., 2019[10]). Both adults and children can develop obesity, but preschool and adolescence periods are thought to be at the highest risk of obesity development (Dereń et al., 2018[24]). Generally, an etiology of obesity may be both genetic and acquired (Laclaustra et al., 2007[68]; Vaiserman et al., 2018[133]; Bayliak et al., 2019[10]).

The amount of fat in the body is controlled by two parallel processes - lipogenesis and lipolysis. When lipogenesis is balanced with lipolysis, the body maintains a constant fat mass. Overweight is developed when lipogenesis dominates over lipolysis, that leads to excessive fat accumulation in the adipose tissue (Laclaustra et al., 2007[68]; Bray et al., 2017[13]; Bayliak et al., 2019[10]). In mammals, there are two types of adipose tissue, namely brown and white one. Brown adipose tissue predominates in newborns and functions to dissipate energy in the form of heat by uncoupling the mitochondrial respiratory electron transport chain from oxidative phosphorylation resulting in the production of heat instead of ATP (Van Eenige et al., 2018[134]). White adipose tissue is the main tissue accumulating storage fats in the form of triacylglycerides (TAG). White adipose tissue is found in subcutaneous and visceral compartments with the latter being an important endocrine organ producing various hormones involved in the regulation of satiety, insulin sensitivity and inflammation (Trayhurn and Wood, 2004[132]; Coelho et al., 2013; Bayliak and Abrat, 2020[9]).

Overweight and obesity lead to a number of metabolic complications collectively called as metabolic syndrome. Clinical diagnosis of metabolic syndrome includes features as follows: increased waist circumference or so-called abdominal obesity, reduced high density lipoprotein level (or “good” cholesterol), elevated blood pressure, and increased blood glucose and TAG levels (Grundy, 2004[47]; Saklayen, 2018[112]). When patients have three or more of these signs, they are diagnosed with metabolic syndrome (Saklayen, 2018[112]). It should be noted that there is debate about causal relationship between obesity and metabolic syndrome: whether obesity is a factor leading to metabolic syndrome or the syndrome causes obesity. Nevertheless, research suggests that excessive fat accumulation, especially in visceral tissue, increases the risk of metabolic syndrome and its various complications, including fatty liver, pro-inflammatory state and oxidative stress, hypertension, dyslipidemia, atherosclerosis, insulin resistance and type 2 diabetes (Grundy, 2004[47]; Laclaustra et al., 2007[68]; Matsuda and Shimomura, 2013[87]; Gastaldelli et al., 2017[39]; Marušić et al., 2021[85]; Bayliak and Abrat, 2020[9]). 

### Short overview of molecular mechanisms of obesity and related metabolic complications 

**Figure 1:**
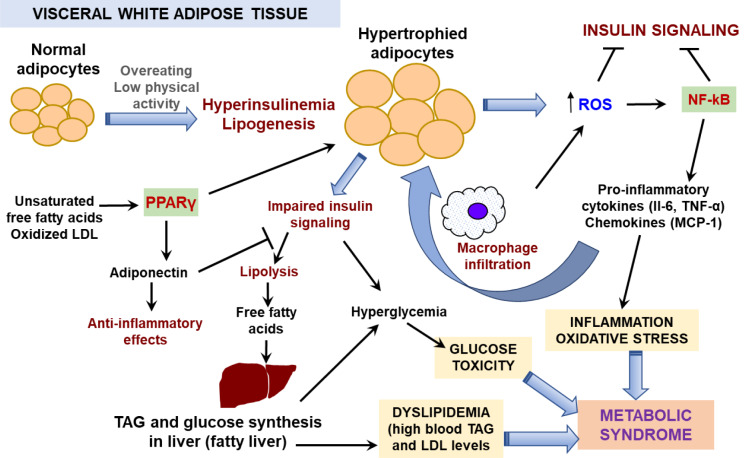
Mechanisms of development of obesity and metabolic syndrome Chronic excessive energy intake especially in combination with low physical activity results in a positive energy balance that may increase insulin production with stimulation of lipogenesis in visceral white adipose tissue. Adipose tissue primarily responds to the higher demand for energy storage by increasing the size of adipocytes leading to adipocyte hypertrophy. As a first step, adipocytes produce higher ROS levels due to stimulation of glucose oxidation via mitochondrial respiration. Increased ROS levels induce activation of adipose AP-1 transcription factor, a regulator of adipose cell proliferation and differentiation, and NF-κB transcription factor, which trigger an acute inflammation response followed by releasing pro-inflammatory mediators, including interleukins (IL-6, IL-1β), TNF-α and MCP-1. The latter mediates macrophage infiltration in adipose tissue. Recruited macrophages produce high levels of ROS as a part of their protective function resulting in intensification of oxidative stress which further stimulates inflammation processes, forming a vicious cycle. Both chronic oxidative stress and inflammation lead to metabolic complications. NF-κB acts as a negative regulator of peroxisome proliferator activated receptor gamma PPARγ, which regulates expression of anti-inflammatory mediators and adiponectin. Adiponectin sensitizes adipocytes to insulin and stimulates lipogenesis. In the hypertrophied adipocytes, synthesis of adiponectin is decreased and that impairs the insulin signaling and stimulates lipolysis with the release of free fatty acids (FFAs) into the bloodstream. FFAs are adsorbed by liver and muscle causing a decrease in insulin sensitivity of these tissues. As a result, blood glucose level increases. Activated gluconeogenesis in insulin-insensitive liver also contributes to hyperglycemia. Liver increases synthesis and accumulation of triacylglycerols (TAG) that impairs its function and provokes atherogenic blood dyslipidemia. Together these processes characterize metabolic syndrome development.

Excessive food consumption can lead to excess nutrients being deposited in adipose tissue in the form of storage lipids, TAG (Figure 1(Fig. 1)). Insulin acts on adipose tissue by sti-mulating of glucose uptake and TAG synthesis with simultaneous suppression of TAG hydrolysis. Fat accumulation is accompanied by adipocyte enlargement up to 10 times in vo-lume. Hypertrophied adipose tissue secretes different active compounds known collectively as adipokines that include chemokines, pro-inflammatory cytokines, and molecules acting as appetite regulators (leptin), insulin sensitizers and atheroprotectors (adiponectin) (Coelho et al., 2013[20]; Smith and Kahn, 2016[123]). In this group, only adiponectin is “a good guy” since it acts to prevent atherosclerotic vascular changes, but its synthesis is gradually decreased with obesity progression (Coelho et al., 2013[20]). Altered insulin sensitivity decreases levels of the major insulin-regulated glucose transporter, GLUT4, in the cell membrane of adipocytes and activates hormone sensitive lipase in adipocytes (Goldberg, 1996[44]). This, in turn, reduces adipose tissue glucose uptake and *de novo* lipogenesis and stimulates lipolysis (Smith and Kahn, 2016[123]). The plasma concentration of free fatty acids (FFAs) increases via lipolysis in adipose tissue, especially from visceral fat (Langin et al., 2006[69]). 

Circulating FFAs are accumulated ectopically in insulin-sensitive tissues and impair insulin action (Morigny et al., 2016[92]). In muscle, increased diacylglycerol levels were shown to stimulate phosphorylation of the serine residues in insulin receptors leading to muscle insensitivity to insulin and suppression of the insulin signaling pathway (Shulman, 2000[120]). In liver, excess FFAs leads to enhanced TAG synthesis that provokes insulin resistance as found in muscles. Increased gluconeogenesis and elevated production of glucose are consequences of reduced insulin effects in the liver. As the result, liver releases more glucose into circulation but due to insulin-insensitivity of muscle it increases blood glucose level (Morigny et al., 2016[92]; Marušić et al., 2021[85]). Hyperglycemia is a clinical sign of insulin dysfunction (Grundy, 2004[47]; Morigny et al., 2016[92]). To combat hyperglycemia, the pancreas secretes more insulin but this does not rescue the situation. Intense TAG biosynthesis with concomitantly suppressed lipogenesis impairs liver functions and causes blood atherogenic dyslipidemia (Grundy, 2004[47]; Marušić et al., 2021[85]).

Macrophage count is also increased in hypertrophied adipose tissue. Increased levels of FFAs, cholesterol, and bacterial lipopolysaccharide (LPS) are well-known inductors of macrophage recruitment. Activated macrophages secrete cytokines and chemokines, such as MCP-1, which, in turn, augment the recruitment of more monocytes and other leukocytes into adipose tissue (Coelho et al., 2013[20]). Increased production of pro-inflammatory cytokines such as tumor necrosis factor alpha (TNF-α) and interleukins 1β (IL-1β) and 6 (IL-6) leads to a low-grade chronic systemic inflammation in adipose tissue and other peripheral tissues (Catrysse et al., 2017[16]; Bayliak and Abrat, 2020[9]). 

Inflammation increases production of reactive oxygen species (ROS) by macrophages as a part of the immune response (Matsuda and Shimomura, 2013[87]). Overloading of mitochondria with energy substrates and activation of adipose NADPH oxidase also contribute to enhanced ROS production followed by oxidative stress development in enlarged adipocytes (Fernández-Sánchez et al., 2011[35]; Kanda et al., 2011[58]; Matsuda and Shimomura, 2013[87]). Experimental data show that enhanced ROS levels stimulate proliferation of pre-adipocytes and increase the size of differentiated adipocytes (Kanda et al., 2011[58]). High blood glucose increases glucose autoxidation and non-enzymatic glycosylation of proteins and nucleic acids; reactive carbonyl species formed by these processes aggravate oxidative damage (Masania et al., 2016[86]; Semchyshyn, 2014[118], 2021[117]; Garaschuk et al., 2018[38]). Increased levels of products of lipid peroxidation, protein and DNA oxidation are observed in blood and adipose tissue of obese patients and mice (Furukawa et al., 2004[37]; Tinahones et al., 2009[131]). Oxidized low-density lipoproteins elicit a strong activation of the nuclear receptor peroxisome proliferator-activated receptor gamma (PPARγ) (Nagy et al., 1998[94]; Zhong et al., 2015[151]), that is one of the main regulators of adipogenesis and lipid storage (Ma et al., 2018[81]; Guo et al., 2020[48]). Under different stimuli or pathological conditions, PPARγ is able to form distinct transcription complexes with different cofactors and transcription partners to express pleiotropic or even opposite effects (Ma et al., 2018[81]; Hernandez-Quiles et al., 2021[52]). In white adipose tissue, activated PPARγ induces an adipogenic program via increasing expression of lipogenic genes including genes encoding fatty acid synthase and cluster of differentiation 36 (CD36) protein, a transmembrane transporter of long chain fatty acids (Ma et al., 2018[81]; Maréchal et al., 2018[84]; Yu et al., 2021[144]). Thus, PPARγ lowers circulating FFA levels and ameliorates lipotoxicity. At the same time, PPARγ enhances insulin sensitivity in adipose and muscle via production of adiponectin which improves glucose utilization, reduces levels of inflammatory cytokines and lowers the intensity of oxidative stress (Dubois et al., 2017[28]; Yanai and Yoshida, 2019[142]). 

Obesity-related increments in ROS production leads to activation of several redox-sensitive transcriptional regulators, including the nuclear factor-κB (NF-κB), activator protein-1 (AP-1), nuclear factor erythroid 2 related factor 2 (Nrf2), and FOXO1 (forkhead box O protein 1) (Fernández-Sánchez et al., 2011[35]; Klotz et al., 2015[63]; Bayliak et al., 2019[10]; Bayliak and Abrat, 2020[9]; Lushchak, 2021[78]). NF-κB and AP-1 induce pro-inflammatory cytokine synthesis and release (Catrysse et al., 2017[16]; Fernández-Sánchez et al., 2011[35]; Bayliak et al., 2019[10]), whereas Nrf2 and FOXO1 are involved in the activation of antioxidant defense and regulation of adipocyte differentiation (Klotz et al., 2015[63]; Bayliak et al., 2019[10]; Lushchak, 2021[78]). Сhronic oxidative stress leads to depletion of antioxidant defenses and activation of a pro-adipogenic program (Klotz et al., 2015[63]; Bayliak et al., 2019[10]). Generally, chronic oxidative stress and related chronic inflammation seem to be the two main interconnecting players in obesity-related metabolic complications.

## Overview of Traditional and Current Medical Use of Chamomile

Chamomile is an annual plant from the family *Asteraceae* or *Compositae* that originates from Southern and Eastern Europe. This herbaceous plant is characterized by a branched erect stem with long and slender leaves and paniculated flower heads consisting of white ray florets and yellow disc florets. Chamomile flowers possess a broad range of pharmaceutical and pharmacological properties due to which the plant has been included into the pharmacopoeia of 26 countries (Singh et al., 2011[121]). In folk medicine, chamomile is used to treat digestion, gynecological and skin problems, eye and mouth infections and is also used as a sedative and analgetic remedy (Singh et al., 2011[121]; Srivastava et al., 2010[124]). Chamomile essential oils are widely used in cosmetology, aromatherapy and even perfumery (Singh et al., 2011[121]). Numerous *in vitro* and *in vivo* studies have demonstrated wound healing, anti-inflammatory, and antimicrobial properties of chamomile preparations. Beneficial effects of chamomile for digestion problems and nervous system functioning were also established (McKay and Blumberg, 2006[88]; Sharifi-Rad et al., 2018[119]). In particular, chamomile preparations are recommended for stress and mental disorders, insomnia, anxiety and hysteria (Amsterdam et al., 2009[4]; Mao et al., 2016[83]).

The most commonly used forms of herbal preparations from chamomile flowers are water decoctions and liquid water-ethanol extracts, while dried extracts in tablet form or as a supplement to creams are used less frequently (European Medicines Agency, 2015[29]). Hot water and alcohol (ethanol or methanol) are the most often used solvents for extraction of biologically active substances from chamomile flowers. There are standardized chamomile preparations containing defined concentrations of marked substances (e.g., api-genin in the concentration of 1.2 %), and non-standardized preparations such as teas or decoctions which contain almost no free apigenin, but contain apigenin glycosides (Srivastava et al., 2010[124]). Herewith, the medicinal properties and chemical composition of chamomile preparations significantly depend on the cultivation conditions of the plant (Singh et al., 2011[121]; Raal et al., 2012[106]). 

## Chemical Composition of Chamomile Flowers

**Figure 2:**
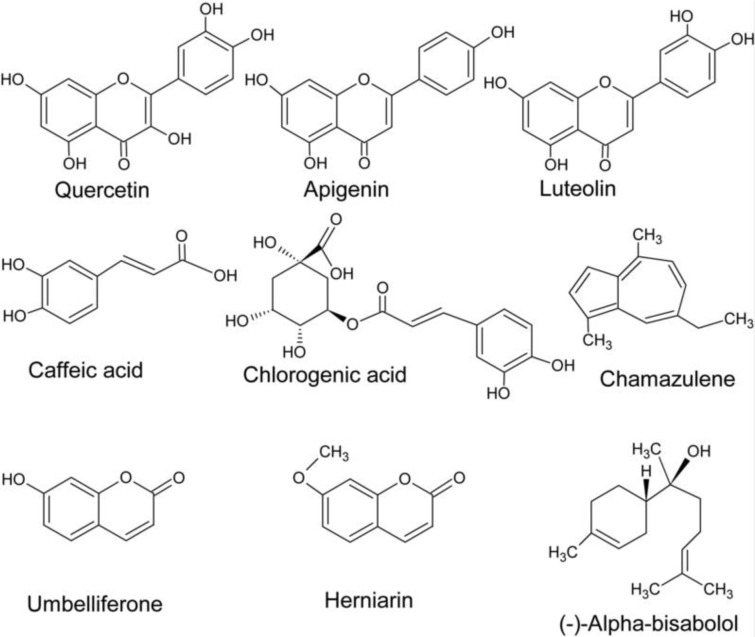
The main constituents of chamomile flowers

Over 100 compounds have been identified in chamomile herb. Chamomile flowers contain essential oils and other terpenes, phenolic compounds (flavonoids, coumarins, and phenolic acids), organic acids, polysaccharides, sterols and mineral compounds (McKay and Blumberg**,** 2006[88]; European Medicines Agency, 201[29]5) (Figure 2(Fig. 2)). The content of different compounds in the plant depends on genetic, ontogenetic and environmental factors (Raal et al., 2012[106]; Ghasemi et al., 2016[43]). Dried chamomile flowers contain up to 2 % of essential oils (McKay and Blumberg, 2006[88]), up to 50 % of phenolic substances (Haghi et al., 2014[50]), and 10-26 % of minerals among which K, Ca, Mg are present in the highest concentrations (McKay and Blumberg, 2006[88]). Other organic acids and polysaccharides make up to 10 % of dry flower mass (McKay and Blumberg, 2006[88]). Among sterols, stigmasterol and β-sitosterol were isolated from chamomile flowers (Ahmad and Misra, 1997[1]).

The main components of essential oils are sequiterpenes and spiroethers. Sequiterpenes include mainly azulenes (1-15 %) with chamazulene being the most abundant, α-bisabolol and its oxides (up to 78 %), and trans-β-farnesene (12-28 %) (Kazemi, 2015[60]; Stanojevic et al., 2016[125]). Flowers also contain non-violate proazulenes (sesquiterpene lactones) like matricin (0.03-0.20 %) and matricarin, that can be partially converted during steam distillation into violate chamazulene of blue color (Capuzzo et al., 2014[15]; European Medicines Agency, 2015[29]; Ghasemi et al., 2016[43]). Bisabolol, a monocyclic sequiterpene alcohol, and bisabol oxides A-D are main constituents providing the aroma of essential oils (Sharifi-Rad et al., 2018[119]). Bisabolol is present in other plants but in chamomile its amounts are the highest (Fernandes et al., 2019[34]). Trans-β-farnesene is involved in chemical communication between plants and protects chamomile from pests (Su et al., 2015[126]). Chamomile spiroethers are presented by cis- and trans-en-in-dicycloethers (up to 20 %) (Redaelli et al., 1981[109]; McKay and Blumberg, 2006[88]). Spiroethers are lipophilic compounds and are important for the quality assessment of chamomile and chamomile extracts, but they are easily decomposed, especially by heating (Redaelli et al., 1981[109]). Minor components of essential oils include α- and β-caryophyllene, caryophyllene-oxide, spathulenol, and some monoterpenes like β-phellandrene, limonene, β-ocimene, γ-terpinen, camphene, sabinene, limonene, 1,8-cineole, camphor, α-pinene, etc. (Kazemi, 2015[60]; Stanojevic et al., 2016[125]; Sharifi-Rad et al., 2018[119]).

Flavonoids and their glycosides, coumarins and phenolic acids are the main groups of phenolic compounds in chamomile flowers (Mulinacci et al., 2000[93]; Singh et al., 2011[121]; Viapiana et al., 2016[135]). The flavonoid group (up to 6 %) includes naringenin (flavanone), quercetin and rutin (flavonols), luteolin and luteolin-7-O-glucoside (flavones), apigenin and apigenin-7-O-glucoside (Singh et al., 2011[121]; Haghi et al., 2014[50]; European Medicines Agency, 2015[29]). The latter is present in high amounts (up to 17 % of total flavonoids) and is used for standardization of chamomile preparations (Miguel et al., 2015[90]). The tannin content in chamomile is less than 1 % (Singh et al., 2011[121]). The coumarins (up to 0.1 %) are mainly represented by herniarin and umbelli-ferone (in a ratio of 1: 5) (Redaelli et al., 1981[109]; Kotov et al., 1991[65]), whereas chlorogenic, caffeic and ferulic acids are major phenolic acids in chamomile flowers (Viapiana et al., 2016[135]). 

## Pharmacological Effects of Chamomile Preparations

The main biological and pharmacological properties of chamomile include antioxidant (Cemek et al., 2008[18]; Capuzzo et al., 2014[15]; Kolodziejczyk-Czepas et al., 2015[64]; Stanojevic et al., 2016[125]; Jabri et al., 2017[55], 2020[54]), anti-inflammatory (Bhaskaran et al., 2010[12]; Miguel et al., 2015[90]; Cavalcante et al., 2020[17]), antimicrobial (Kazemi, 2015[60]; Stanojevic et al., 2016[125]), antispasmodic (Albrecht et al., 2014[2]; Gentile et al., 2018[40]) and sedative (Amsterdam et al., 2009[4]) activities.

The antioxidant properties of chamomile are attributed mainly to its phenolic substances especially flavonoids (apigenin, luteolin, quercetin, etc.) and their glycosides (McKay and Blumberg, 2006;[88] de Franco et al., 2020[21]), although chamomile essential oils also possess antioxidant activity (Capuzzo et al., 2014[15]; Stanojevic et al., 2016[125]; Wang et al., 2020[139]). Due to the presence of active hydroxyl groups, phenols can serve as donors of electrons and scavenge free radicals and reduce oxidized biomolecules. Phenols with higher numbers of hydroxyl groups have greater electron donor activity and, respectively, higher antioxidant properties (Foti, 2007[36]). In glycosylated form, phenolic compounds usually exhibit lower antioxidant activity (de Franco et al., 2020[21]). The chamomile coumarins, herniarin and umbelliferone, being phenolic substances also have an ability to quench electron exciting molecules, in particular those formed by ultraviolet light. Due to the ability of coumarins to strongly absorb ultraviolet rays, chamomile extracts are used in the cosmetic field for skin defense (Molnar et al., 2017[91]). It is possible that recorded *in vivo* antioxidant effects of phenolic substances are also connected with their effects as weak prooxidants due to which they up-regulate expression of Nrf2-controlled antioxidant enzymes (Lushchak, 2021[78]).

The anti-inflammatory effects of chamomile preparations or their isolated compounds have been identified in studies of multiple experimental models (Miguel et al., 2015[90]; Wang et al., 2020[138]; Cavalcante et al., 2020[17]) and when used to treat various diseases associated with the development and progression of inflammatory processes (Jabri et al., 2020[54]; Wang et al., 2020[138]). For example, treatment of macrophages with lipopolysaccharide (LPS) from *Escherichia coli is *a well-established experimental system for studies of inflammation and stimulates a range of signaling pathways, including NF-κB-mediated signaling that enhances the transcription of mRNA encoding pro-inflammatory cytokines such as IL-1β, IL-6, TNF-α (Tanaka et al., 2014[130]). The incubation of macrophages with LPS in the presence of polyphenol-rich chamomile extracts or apigenin-7-O-glucoside decreased production of pro-inflammatory cytokines IL-1β, IL-6 and TNF-α (Drummond et al., 2013[27]; Miguel et al., 2015[90]). Apigenin-7-O-glucoside also had strong inhibitory effects on LPS-induced NF-κB/NLRP3/caspase-1 signaling in macrophage RAW246.7 cells (Wang et al., 2020[138]). Moreover, apigenin-7-O-glucoside was found to suppress LPS-induced mRNA expression of iNOS and cyclooxygenase-2 (COX-2), lowering release of inflammatory mediators, ^•^NO and prostaglandin E2 (PGE2) (Hu et al., 2016[53]). Such effects are likely to be due to blocking the activation of the pro-inflammatory signaling cascade mediated by NF-κB (Kim et al., 2011[62]; Bhaskaran et al., 2010[12]; Ma et al., 2020[80]). In addition to flavonoids, components of essential oils like bisabolol, chamazulene and farnesene were shown to possess anti-inflammatory properties (Cavalcante et al., 2020[17]; Wang et al., 2020[139]).

## Сhamomile in the Management of Metabolic Syndrome and Obesity

### Anti-hyperglycemic and anti-hyperlipidemic effects of whole chamomile extracts 

Administration of ethanolic extract from chamomile flowers decreased postprandial hyperglycemia and oxidative stress intensity and augmented the antioxidant system in rats with streptozotocin (STZ)-induced diabetes (Table 1(Tab. 1); References in Table 1: Al-Jubouri et al., 1990[3]; Cemek et al., 2008[18]; Feng et al., 2017[33]; Gentile et al., 2018[40]; Jabri et al., 2017[55], 2020[54]; Jung et al., 2016[57]; Kato et al., 2008[59]; Khan et al., 2014[61]; Lv et al., 2019[79]; Najla et al., 2012[95]; Panda et al., 2007[99]; Rafraf et al., 2015[107]; Rauter et al., 2010[108]; Weidner et al., 2013[140]; Wu et al., 2021[141]; Yang et al., 2018[143]; Zemestani et al., 2016[146]). As a result, the extract exhibited a strong antihyperglycemic effect and protected beta-cells of pancreas against ROS in diabetic rats (Cemek et al., 2008[18]). Chamomile hot water extract (chamomile tea) showed moderate suppression of hyperglycemia in a sucrose-loading test and a STZ-induced rat diabetes model (Kato et al., 2008[59]). In addition, chamomile water extract reduced the elevated fasting blood glucose in STZ-induced diabetic rats (Najla et al., 2012[95]).

Aqueous chamomile extracts reduced serum cholesterol levels in hyperlipidemic rats but had no effect on serum triacylglyceride levels (Al-Jubouri et al., 1990[3]). Another study showed that chamomile decoction extract prevented the increase in body mass, oxidative stress development in liver and kidney, and disturbances in blood lipid profile in rats fed with high fat diet (Jabri et al., 2017[55]). Neuroprotective effects of chamomile decoction were also observed in rats on a high fat diet (Jabri et al., 2020[54]). Feeding with high fat diet induced the anxiogenic-like symptoms, inflammation and ROS production in rat brain, and these changes were alleviated by administering of chamomile extract (Jabri et al., 2020[54]). These findings together confirm potential anti-obesity and anti-lipidemic properties of chamomile preparations.

Some studies suggest that chamomile preparations can prevent metabolic overload effects of high glucose or fructose diets by attenuating their absorption through inhibition of carbohydrate-digestive enzymes and hexose transporters in the intestine (Villa-Rodriguez et al., 2018[136]). The inhibition of key enzymes involved in gluconeogenesis and glycogenolysis as well as stimulation of glucose utilization (mostly in muscle and adipose tissue) is considered to contribute to chamomile-induced changes of blood glucose levels (Kato et al., 2008[59]; Rafraf et al., 2015[107]; Zemestani et al., 2016[146]). Apigenin, apigenin-7-*O*-glucoside, and (*Z*) and (*E*)-2-hydroxy-4-methoxycinnamic acid glucosides were identified as the most active polyphenols in chamomile flowers that may modulate carbohydrate digestion and absorption *in vitro* as assessed by inhibition of activities of α-amylase and maltase (Villa-Rodriguez et al., 2018[136]). 

The treatment of insulin-resistant high-fat diet fed C57BL/6 mice with chamomile ethanol extract considerably reduced insulin resistance, glucose intolerance, and levels of plasma TAG, non-esterified fatty acids and low density cholesterol (Weidner et al., 2013[140]). Chamomile improves insulin sensiti-vity via affecting the peroxisome proliferator-activated receptor (PPAR) family, a group of transcription factors that participate in glucose and lipid homeostasis. Modulation of PPARs is commonly used for insulin resistance and dyslipidemia treatment (Dubois et al., 2017[28]). Chamomile extract was shown to activate the nuclear receptor PPARγ in human primary adipocytes leading to specific expression of PPARγ target genes (Weidner et al., 2013[140]). 

Administration with chamomile tea reduced body mass gain, fasting and postprandial blood glucose levels and levels of glycated hemoglobin in alloxan-induced diabetic rats, and these results were comparable with glibenclamide used as an anti-diabetic standard (Khan et al., 2014[61]). A single-blind randomized controlled clinical trial carried out with 64 individuals with type 2 diabetes (males and females) showed that chamomile tea consumed three times per day significantly decreased concentrations of glycated hemoglobin and serum insulin, total cholesterol, triacylglyceride and low-density lipoprotein cholesterol (Rafraf et al., 2015[107]). Patients also showed decreased serum malondialdehyde levels and higher serum total antioxidant capacity as compared with the control group (Zemestani et al., 2015[146]). Such effects might be associated with reduction of insulin resistance.

### Effects of individual chamomile constituents 

In this part of the paper, we analyze anti-obesity and anti-diabetic effects of some chamomile constituents - the flavonoids apigenin and luteolin and the sesquiterpenes α-bisabolol and chamazulene. Сhamomile flowers contain other important compounds like quercetin or ferulic acid but they are analyzed in detail elsewhere (Pérez-Torres et al., 2021[103]). 

#### Apigenin

Marker compounds of chamomile preparations apigenin and its glycoside apigenin-7-O-glucoside have been well-studied for their antioxidant, anti-inflammatory and antimicrobial effects (Gentile et al., 2018[40]; Wang et al., 2019[137]; Salehi et al., 2019[113]). In addition, recent studies demonstrated effectiveness of apigenin in the prevention and treatment of obesity-related metabolic complications (Feng et al., 2017[33]; Gentile et al., 2018[40]; Yang et al., 2018[143]; Lv et al., 2019[79]) (Table 1(Tab. 1)). 

Oral administration of apigenin (0.78 mg/kg body weight) for 10 days was reported to reverse the decrease in hepatic antioxidant potential in insulin-dependent diabetic mice (Panda and Kar, 2007[99]). Intraperitoneal administration of apigenin had a significant antihyperglycemic effect in rats with streptozotocin-induced diabetes (Rauter et al., 2010[108]). In cloned pancreatic β-cells, apigenin treatment attenuated 2-deoxy-D-ribose-induced apoptosis due to its antioxidant activity and controlling the mitochondrial membrane potential (Suh et al., 2012[129]). In monotypic human THP-1 cells, apigenin inhibited the activation of NF-κB-induced synthesis of cytokines TNF-α and IL-1β (Zhang et al., 2014[148]). In HepG2 hepatocytes treated with palmitic acid, the flavonoid reduced excessive lipid accumulation, enhanced phosphorylation of AMP-activated protein kinase (AMPK) and decreased levels of 3-hydroxy-3-methylglutaryl CoA reductase (Lu et al., 2019[75]). Apigenin also prevented lipid accumulation in HepG2 cells exposed to high glucose levels, and these effects were accompanied by increased phosphorylation of AMPK and its downstream target, acetyl-CoA carboxylase (Zang et al., 2006[145]), confirming that apigenin has a beneficial effect on dyslipidemia and diabetes by regulating energy metabolism.

In mice fed a high fat diet (HFD), apigenin counteracted the increase in body and epididymal fat mass and prevented alterations of metabolic and inflammatory parameters (Gentile et al., 2018[40]). In addition, apigenin decreased levels of malondialdehyde, IL-1β and IL-6 as well as expression of iNOS in the colon of HFD mice, preventing enteric inflammation and normalizing colonic dysmotility associated with obesity (Gentile et al., 2018[40]). Another study showed that gavage administration of apigenin mitigated HFD-induced liver injury, enhanced insulin sensitivity and reduced lipid accumulation in mouse liver (Lv et al., 2019[79]). Similar results were obtained by Jung et al. (2016[57]) that showed ameliorative effects of apigenin on blood lipid profile, insulin resistance and hepatic steatosis in obese mice fed with HFD. Importantly, apigenin reversed the HFD-induced activation of the NLRP3 inflammasome with a further reduction in the production of pro-inflammatory cytokines IL-1β and IL-18, the inhibition of xanthine oxidase activity and the reduction of uric acid and ROS production (Lv et al., 2019[79]). Thus, apigenin restrained progression of non-alcoholic fatty liver development in mice fed with HFD. The hepatoprotective effects of apigenin were connected with upregulation of expression of genes involved in fatty acid oxidation, tricarboxylic acid cycle, and oxidative phosphorylation, and down-regulation of expression of lipolytic and lipogenic genes (Jung et al., 2016[57]). Modulation of PPARγ activity was found to be responsible for metabolic and transcriptional changes in apigenin-treated HFD mice (Feng et al., 2016[32], 2017[33]; Pallauf et al., 2017[98]). In adipose tissue of HFD mice, apigenin activated PPARγ and blocked translocation of a complex of PPARγ and p65 (the active subunit of NF-κB factor) into the nucleus, thereby decreasing NF-κB activation and eventually inhibiting inflammation (Feng et al., 2016[32]). In HFD mouse liver, apigenin had no obvious activating effects on PPARγ but significantly inhibited the expression of PPARγ target genes encoding proteins associated with lipid metabolism (Feng et al., 2017[33]). At the same time, apigenin caused activation of the transcription factor Nrf2, which translocated into the nucleus and affected expression of genes whose products could inhibit lipid metabolism and increase antioxidant defense (Feng et al., 2017[33]). It was suggested that activation of Nrf2 may counteract PPARγ activation in mice fed with HFD and apigenin (Feng et al., 2017[33]). 

In addition to its protective effects against HFD, administration of apigenin showed beneficial effects via improvement of insulin resistance, alleviation of liver injury, and inhibition of the alterations in lipid profiles in high fructose-fed mice (Yang et al., 2018[143]). These effects were accompanied by translocation and nuclear accumulation of Nrf2 followed by increasing expression of heme oxygenase-1 and NAD(P)H:quinone acceptor oxidoreductase-1 genes involved in protection against ROS (Yang et al., 2018[143]). In human HepG2 cells, apigenin induced translocation of the FOXO1 transcription factor (an important mediator of insulin signal transduction) into the nucleus via inhibition of the PKB/AKT-signaling pathway (Bumke-Vogt et al., 2014[14]). Inhibition of expression of gluconeogenic and lipogenic genes was also found in apigenin-treated HepG2 cells and this was accompanied by activation of the Nrf2 signaling pathway, suggesting that the latter is involved in the inhibition of expression of gluconeogenic enzymes (Bumke-Vogt et al., 2014[14]). 

No anti-adipogenic action was found for apigenin when human pre-adipocytes (mesenchymal fat cells) were treated with this flavonoid (Gómez-Zorita et al., 2017[45]). However, apigenin prevented TAG accumulation in mature adipocytes by activating lipolysis (Gómez-Zorita et al., 2017[45]). It has been reported recently that polyphenols and phenolic acids could inhibit adipogenesis in mouse 3T3-L1 preadipocytes with different intensity depending on the compound and the stage of adipocyte differentiation (Aranaz et al., 2019[6]). Ferulic and gallic acids were active only at the early stages of differentiation, quercetin and resveratrol inhibited lipid accumulation along the whole process of differentiation, whereas apigenin was active during the early and latest stages (Aranaz et al., 2019[6]). Apigenin-7-O-glucoside also inhibited adipogenesis of 3T3-L1 preadipocytes at the early stage of adipogenesis by downregulation of PPARγ (Hadrich and Sayadi, 2018[49]). Based on molecular docking analysis, it was proposed that phenolic compounds interact with specific residues of the PPARγ receptor, which could determine their potential anti-adipogenic activity during the early stages of differentiation (Aranaz et al., 2019[6]). 

In addition to PPARγ, the signal transducer and activator of transcription 3 (STAT3) is another important molecular player in obesity. The activity of STAT3 is elevated in visceral adipocytes of obese subjects and is considered as a pathogenic factor of visceral obesity (Priceman et al., 2013[104]). HFD increases the phosphorylation of STAT3 that leads to STAT3 nuclear translocation and its enhanced transitional activity in visceral adipose tissue (Su et al., 2020[127]). Apigenin was found to bind to non-phosphorylated STAT3 and thus reduced STAT3 phosphorylation and expression of target genes, including CD36 gene, in adipocytes (Su et al., 2020[127]). The product of the CD36 gene was first identified as a transmembrane fatty acid transporter involved in long chain fatty acid uptake in multiple tissues but is now recognized as a multi-functional receptor (Maréchal et al., 2018[84]). In particular, the role of CD36 in regulation of PPARγ downstream actions with benefits for hepatic lipid metabolism was demonstrated (Maréchal et al., 2018[84]; Yu et al., 2021[144]). Inhibition of adipocyte differentiation under treatment with apigenin was accompanied by decreased expression of both CD36 and PPARγ; however, overexpression of STAT3 reversed the apigenin-inhibited adipogenesis (Su et al., 2020[127]). However, it remains controversial whether the reduced expression of PPARγ resulted from decreased CD36 signaling (Maréchal et al., 2018[84]) or reduced PPARγ activity lowered CD36 expression (Lim et al., 2006[72]; Yu et al., 2021[144]). Wu et al. (2021[141]) have recently found that apigenin could decrease levels of sterol regulatory element-binding protein 1c (SREBP-1c) and sterol regulatory element-binding protein 2 (SREBP-2) and their downstream genes encoding fatty acid synthase, stearyl-CoA desaturase 1 and 3-hydroxy-3-methyl-glutaryl-CoA reductase in hyperlipidemic HepG2 cells and mice fed with HFD. These findings support antilipidemic properties of apigenin.

### Luteolin and essential oils

Like apigenin, luteolin is a yellow flavonoid and is an active ingredient of chamomile flowers (Ding et al., 2010[26]). Pharmacological effects of luteolin include antioxidant, antitumor, anti-inflammatory and antimicrobial actions (Lopez-Lazaro, 2009[74]). Beneficial effects of luteolin in obesity and insulin resistance have also been reported (Ding et al., 2010[26]; Liu et al., 2014[73]; Bumke-Vogt et al., 2014[14]). Luteolin was found to intensify transcriptional activity of PPARγ in 3T3-L1 adipocytes and increase their insulin sensitivity by up-regulation of the expression of insulin-dependent glucose transporter (GLUT-4) receptors (Ding et al., 2010[26]). Luteolin also enhanced expression of the adiponectin gene, which is one of the PPARγ targets and involved in regulation of glucose levels and fatty acid breakdown (Ding et al., 2010[26]). Adipose tissue secretes adiponectin into the bloodstream. The adiponectin receptors present in liver and muscle cells affect the downstream target, AMP kinase, an important regulator of cellular energy metabolism (Fang and Sweeney, 2006[30]). Metabolic effects of adiponectin include an increase in glucose intake in skeletal muscle and inhibition of glucose production in liver (Lihn et al., 2005[71]; Fang and Sweeney, 2006[30]).

In mice fed with HFD, luteolin attenuated body mass gain (Gentile et al., 2018[41]) and accumulation of epididymal fat and counteracted metabolic alterations in mesenteric vessels, such as changes in relaxation response, increase in TNF-α levels and ROS levels (Gentile et al., 2018[41]). Pre-treatment with luteolin also prevented activation of the NF-κB transcription factor and production pro-inflammatory cytokines in palmitate-stimulated endothelial cells and restored insulin sensiti-vity in the endothelium (Deqiu et al., 2011[23]). Antioxidant and anti-inflammatory mechanisms were assumed to be responsible for preventive effects of luteolin in systemic metabolic alterations and vascular dysfunction associated with obesity (Gentile et al., 2018[41]). Like apigenin, luteolin was found to induce translocation of the FOXO1 transcriptional factor into the nucleus and activate Nrf2 that was accompanied by inhibition of gluconeogenic and lipogenic capacity in human cells (Blumke-Vogt et al., 2014[14]). Several studies demonstrated protective effects of luteolin against obesity-related complications (Liu et al., 2014[73]; Zhang et al., 2016[147][149]; Kwon et al., 2015[67]; Kwon and Choi, 2018[66]; Baek et al., 2019[7]; Park et al., 2020[100]). In particular, dietary luteolin attenuated adipose tissue inflammation and insulin resistance in obese ovariectomized mice (Baek et al., 2019[7]) and prevented hepatic fibrosis and insulin resistance in HFD-fed mice (Kwon et al., 2015[67]; Kwon and Choi, 2018[66]). The protective effects were accompanied by activation of AMPK-signaling (Zhang et al., 2016[147][149]) and lowering of inflammation (Kwon and Choi, 2018[66]; Baek et al., 2019[7]).

Individual essential oils from chamomile flowers have not been studied as anti-obesity agents but some studies reported anti-inflammatory effects of the chamomile sesquiterpenes α-bisabolol and chamazulene in experimental models of inflammation (Rocha et al., 2011[111]; Fernandes et al., 2019[34]; Cavalcante et al., 2020[17]; Ma et al., 2020[80]). In addition, antioxidant activity of α-bisabolol and chamazulene was demonstrated in both *in vitro* and *in* *vivo* studies (Capuzzo et al., 2014[15]; Querio et al., 2018[105]; Wang et al., 2020[139]; Cavalcante et al., 2020[17]). In particular, chamazulene attenuated ROS production in bovine aortic endothelial cells treated with either high glucose or H_2_O_2_ (Querio et al., 2018[105]). Thus, thanks to antioxidant and anti-inflammatory effects, essential oils seem to contribute to the anti-obesity effects of chamomile extracts in experimental models. 

### General picture of mechanisms of chamomile action in obesity-related complications

**Figure 3:**
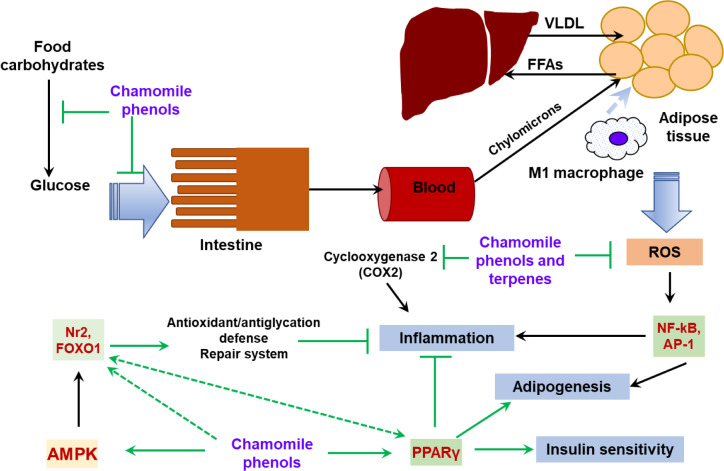
General scheme of potential protective mechanisms of chamomile constituents in obesity Chamomile phenolic compounds, especially flavonoids (apigenin, luteolin and quercetin) and their glycosides are able to slow down food digestion and absorption in the intestine by inhibition of enzymes involved in carbohydrate breakdown (amylase and maltase) and inhibition of transporters for monosaccharides. The chamomile analog of cholesterol sitosterol is supposed to decrease food cholesterol absorption via competitive mechanism. Chamomile phenolic compounds and essential oils possess antioxidant properties; therefore, they demonstrate the ability to protect against oxidative stress related to obesity. Аnti-inflammatory activity of chamomile compounds (dependent and independent one on antioxidant properties) could ameliorate obesity-induced inflammation. Direct modulation of PPARγ activity seems to be another protective mechanism that decreases lipotoxicity and improves insulin resistance of obesity. In addition, chamomile whole extracts and individual phenols can lead to activation of regulators of stress responsive Nrf2 and FOXO1 transcription factors suggesting a hormetic mechanism of the action. Green lines denote possible routes of chamomile action.

Data from the literature described above suggest that chamomile preparations can act against obesity and its complications by different mechanisms. The possible protective mechanisms are summarized in Figure 3(Fig. 3) and include:

**(1) Inhibition of carbohydrate digestion and glucose absorption in the intestine**. These mechanisms may improve glycemic control in obese people and attenuate postprandial hyperglycemia in type 2 diabetics and may also promote loss of body mass (Kato et al., 2008[59]; Rafraf et al., 2015[107]; Zemestani et al., 2016[146]; Villa-Rodriguez et al., 2018[136]). 

**(2) Reduction of blood cholesterol levels**. Chamomile flowers contain β-sitosterol (Ahmad and Misra, 1997[1]) which was found to interfere with cholesterol absorption in the intestine and prevent de novo cholesterol biosynthesis by inhibition of the hydroxy-methylglutaryl coenzyme A reductase and sterol C24‐reductase gene expression in the intestine (Feng et al., 2020[31]).

**(3)Direct antioxidant action of phenolic constituents and essential oils**. Phenolic compounds can augment endogenous antioxidant defenses and help to ameliorate oxidative damage during obesity progression (Jabri et al., 2017[55], 2020[54]).

**(4) Anti-inflammatory action**. Chamomile preparations are connected with the inhibition of synthesis of inflammatory mediators, ^•^NO and prostaglandins, and pro-inflammatory cytokines and chemokines (Kim et al., 2011[62]; Hu et al., 2016[53]; Ma et al., 2020[80]; Wang et al., 2020[138]). Obesity is characterized by development of chronic inflammation with NF-κB being the major contributor. NF-κB as a redox-sensitive transcription factor undergoes activation when ROS levels rise. Attenuation of ROS production in obesity due to the antioxidant activity of chamomile preparations сan contribute to the prevention of NF-κB hyperactivation and the subsequent release of downstream inflammatory cytokines. 

**(5) Modulation of PPARγ activity**. As a gene regulator PPARγ can act via transactivation and transrepression mechanisms. In the case of transactivation, PPARγ forms a complex with the retinoid X receptor (RXR) and ligand binding to PPARγ/RXRα heterodimers promote corepressor release and recruitment of coactivators, leading to initiation of transcription. In the case of transrepression, ligand binding to PPARγ potentiates its repression effect on transcription factors, in particular this mechanism suppresses the NF-κB and AP-1 pro-inflammatory signaling pathways (Hernandez-Quiles et al., 2021[52]). Flavonoid components of chamomile can bind directly to PPARγ and thereby activate this protein promoting its anti-inflammatory effects (Feng et al., 2016[32]). PPARγ may activate expression of the Nrf2 gene and is one of the target genes of Nrf2 signaling (Lee, 2017[70]) but their link in obesity is not obvious. In HFD models, apigenin activated expression of both Nrf2 and PPARγ genes but suppressed expression of PPARγ-dependent genes (Feng et al., 2017[33]).

**(6) Activation of Nrf2 and FOXO stress regulators**. The primary function of Nrf2 action is to maintain redox homeostasis via upregulation of the expression of antioxidant and xenobiotic-detoxifying enzymes (Lushchak, 2011[77], 2021[78]; Zhang et al., 2015[150]). Enhanced Nrf2 activity was shown to inhibit inflammation and prevent adipogenesis and the onset of diabetes mellitus (Slocum et al., 2016[122]). FOXO proteins, in particular FOXO1, also regulate cellular stress responses via induction of expression of defenses (Klotz et al., 2015[63]). In addition, FOXO1 modulates energy homeostasis through regulation of adipocyte size and adipose tissue-specific gene expression (Nakae et al., 2008[96]). As described above, whole chamomile preparations or its individual compounds (apigenin, quercetin, luteolin) are able to activate Nrf2 or FOXO1 and enhance expression of their target genes (Bhaskaran et al., 2010[12]; Bumke-Vogt et al., 2014[14]; Feng et al., 2017[33]; Pallauf et al., 2017[98]). In contrast to antioxidant mechanisms, the activation of redox-sensitive factors Nrf2 and FOXO1 by chamomile phenolic compounds appears to involve a pro-oxidant mechanism. In particular, it was proposed that plant flavonoids like apigenin and quercetin undergo oxidation to quinones, and then either directly or via ROS formation cause dissociation of Nrf2 from its inhibitor Keap1, resulting in nuclear translocation of the Nrf2 factor and leading to expression of target genes (Pallauf et al., 2017[98]). Induction of antioxidant and anti-inflammatory proteins, and proteins regulating energy metabolism could be involved in the protective effects of flavonoid-mediated Nrf2-stimulation in obesity (Feng et al., 2017[33]).

**(7) AMPKactivation.** The AMP-activated kinase is a cellular energy sensor responding to low ATP levels (e.g., as in a state of caloric restriction) and its activation restores energy homeostasis and stimulates FOXO1 and Nrf2 signaling pathways that provide cellular stress resistance and adaptation (Salminen and Kaarniranta, 2012[114]). Activation of AMPK was found in adipose tissue of luteolin-treated mice (Zhang et al., 2016[147][149]) and in HepG2 cells treated with apigenin and palmitate (Lu et al., 2019[75]). It seems that phytochemicals can indirectly activate AMPK by acting as mimetics of caloric restriction or via their pro-oxidant mechanism.

### Efficiency of chamomile preparations

Several human clinical trials have confirmed the effectiveness of chamomile tea in the improvement of inflammatory markers, lipid profile and insulin resistance in patients with type 2 diabetes (Rafraf et al., 2015[107]; Zemestani et al., 2016[146]; Hajizadeh-Sharafabad et al., 2020[51]) (Table 1(Tab. 1)). To our best knowledge, no clinical trials are available regarding chamomile use in the management of obesity. Data from animal studies suggest that chamomile preparations could show potentially moderate improvement in obesity and its related complications. Moderate efficacy could be connected to large extent with a low bioavailability of plant polyphenolic compounds for oral consumption (Manach et al., 2005[82]).

Moreover, this issue can be applied to a number of the polyphenol-containing plants that show good anti-obesity effects *in vitro* in experimental models and efficacy when used in daily human consumption. In animal studies, chamomile preparations or its individual compounds are usually administrated to animals over 6-24 weeks to observe favorable effects. However, several weeks of mouse or rat lifespan do not correspond several weeks of human lifespan. Therefore, it should likely take more time than in rodent models to detect strong positive effects of chamomile preparations in humans. Taking into account the absence of data on chamomile toxicity, long-term consumption of chamomile preparations seems to be plausible to achieve desirable effects. Encapsulation of chamomile extracts or individual flavonoids in biocompatible nanoparticles is one of the perspective approaches to increase bioavailability and efficacy of chamomile preparations in the management of obesity-related complications (Dewanjee et al., 2020[25]), and this issue needs further investigation. 

### Possible risks and adverse effects of chamomile preparations

Chamomile is included in the GRAS (generally recognized as safe) list of the United States Food and Drug Administration (FDA), which means a substance added to food is considered safe by experts (Srivastava et al., 2010[124]). However, some studies report that using of chamomile preparations can cause certain adverse effects. In particular, allergic reactions are the most common adverse effects of chamomile-containing remedies (McKay and Blumberg, 2006[88]; Benito et al., 2014[11]; European Medicines Agency 2015[29]), and the allergies are often observed in people who are allergic to other plant members of the family *Compositae* (de la Torre Morín et al., 2001[22]; Paulsen et al., 2008[101]). Chamomile topical application most often induces contact dermatitis (Anzai et al., 2015[5]), rhinitis (Scala, 2006[115]) and eye irritation and conjunctivitis (Subiza et al., 1990[128]). In addition, hypersensitivity reactions including severe allergic reactions (Quincke's disease, dyspnea, vascular collapse, anaphylactic shock) after mucosal contact with liquid chamomile preparations have been documented (Reider et al., 2000[110]; European Medicines Agency 2015[29]; Nakagawa et al., 2019[97]). People with asthma should take chamomile drugs with caution so as not to exacerbate the disease (European Medicines Agency 2015[29]). Sesquiterpene lactones (Lundh et al., 2007[76]) and coumarin herniarin (Paulsen et al., 2010[102]) are considered as main sensitizers in chamomile preparations. Chamomile coumarins may act as a vitamin K antagonist and interfere with the blood coagulation processes (Segal and Pilote, 2006[116]). 

Due to chamomile mild sedative effects (Chaves et al., 2020[19]), long-term oral consumption of chamomile preparations can suppress the central nervous system (CNS) and potentiate CNS depressant effects of other sedative drugs such as opioid analgesics and alcohol and, therefore, it is recommended not using in conjunction with these agents (McKay and Blumberg, 2006[88]).

Since chamomile essential oils and phenolic compounds possess a strong antibacterial activity, the oral consumption of chamomile preparations at high concentrations can affect gut microbiota composition. In this, context, both beneficial and undesirable gut bacteria can undergo qualitative and quantitative changes resulting in health adverse effects such as gastrointestinal problems. However, future clinical studies on this issue are needed. At high concentrations, plant phenols exert pro-oxidant activity (Bayliak et al., 2016[8]); therefore, their protective action can be suppressed, even opposite effects may occur. Thus, dose-dependency of chamomile effects always should be taken into account. 

## Conclusions

Chamomile flowers contain a wide range of polyphenolic compounds and essential oils that possess various biological activities including antioxidant, anti-inflammatory and energy metabolism modulating effects. Due to these properties chamomile preparations can be effectively used for obesity prevention and treatment. Whole chamomile extract seems to be more effective than isolated individual components since the latter show some differences in cellular and protein targets and together may demonstrate synergetic effects. More research is needed to establish the molecular mechanisms of action of chamomile preparations. In particular, there is a need to further explore the involvement of PPARγ and Nrf2 signaling pathways because these were found to play dual roles in obesity demonstrating both anti-obesity and obesity-promoting effects (Zhang et al., 2015[150]; Ma et al., 2018[81]; Bayliak and Abrat, 2020[9]). 

## Acknowledgements

This work was partially supported by a grant from the Ministry of Education and Science of Ukraine (#0118U003477) to VIL and a grant from National Research Foundation of Ukraine (#2020.02/0118) to MMB.

## Conflict of interest

The authors declare that they have no conflict of interest.

## Authors’ contributions

Maria M. Bayliak provided idea and design of the article, prepared figures and tables, performed review and editing; provided funding acquisition; Tetiana R. Dmytriv collected literature, wrote the original draft and prepared figures; Antonina V. Melnychuk and Nadia V. Strilets collected literature and wrote the original draft; Kenneth B. Storey performed review and editing and provided valuable discussion; Volodymyr I. Lushchak performed analysis, review and editing the manuscript, provided funding acquisition. All authors read and approved the final manuscript.

## Figures and Tables

**Table 1:**
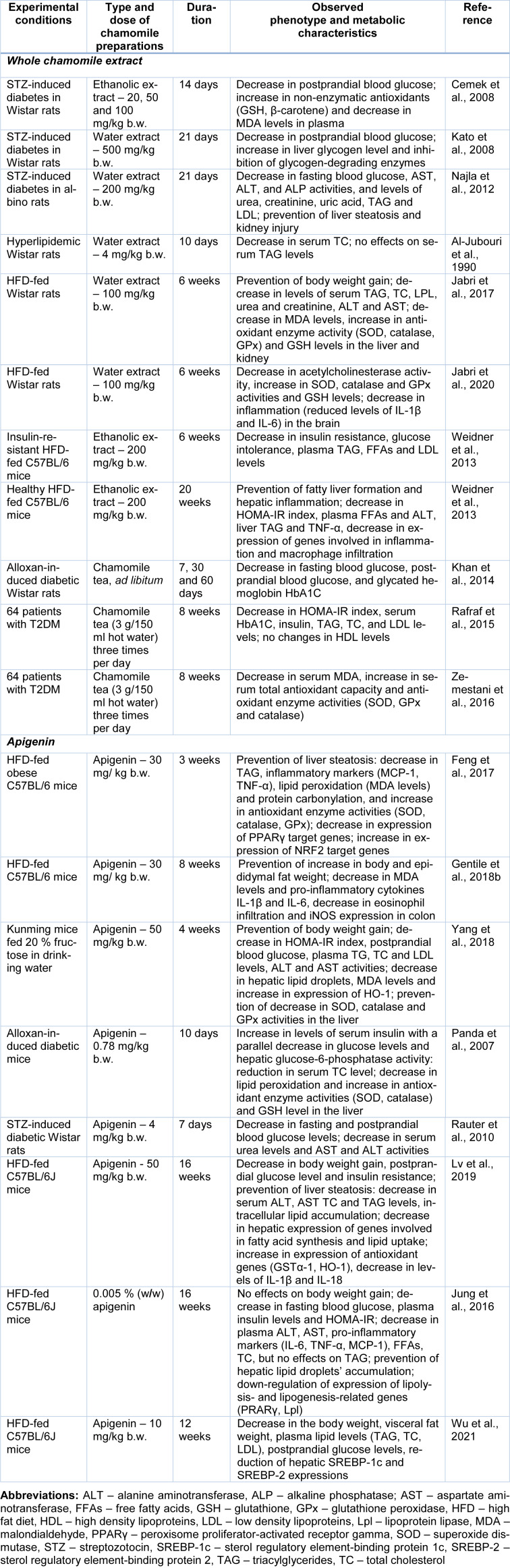
*In vivo* anti-obesity and anti-diabetic activities of whole chamomile flower extracts and apigenin, one of the main chamomile compounds
